# Supplementary material for: Neurons in auditory cortex integrate information within a constrained and context-invariant temporal window
Source: Curr Biol. Author manuscript; Available in PMC 2026 Jul 3. (PMC13331568; doi:10.1016/j.cub.2025.11.011)
Supplement: Supplemental 1 [file NIHMS2179570-supplement-Supplemental_1.pdf]

Current Biology, Volume *35*

Supplemental Information

Neurons in auditory cortex integrate information within a constrained and context-invariant temporal window

Magdalena Sabat, Hortense Gouyette, Quentin Gaucher, Mateo López Espejo, Stephen V. David, Sam Norman-Haignere, and Yves Boubenec

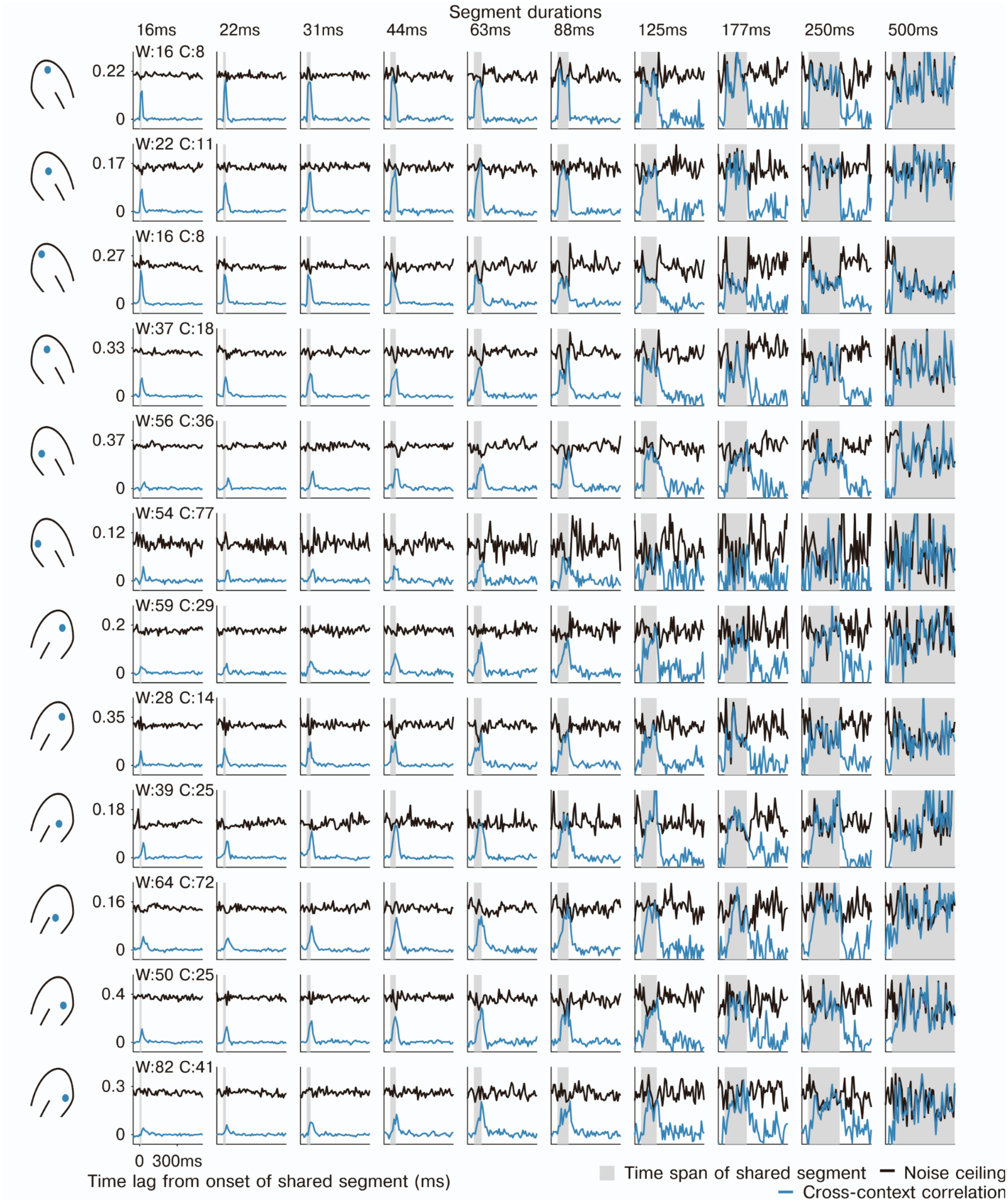

Figure S1. Examples of cross-context correlation for multi-units in different regions of the auditory cortex. Related to Figure 1.

Examples of the CCC (blue) and noise ceiling (black) from example multi-units from primary and non-primary ferret auditory cortex. For all units, there is a lag and segment duration for which CCC equals the noise ceiling, indicating a context-invariant response. The segment duration needed to achieve a

context-invariant response varies substantially across units. The location of each recorded multi-unit is indicated to the left of each row.

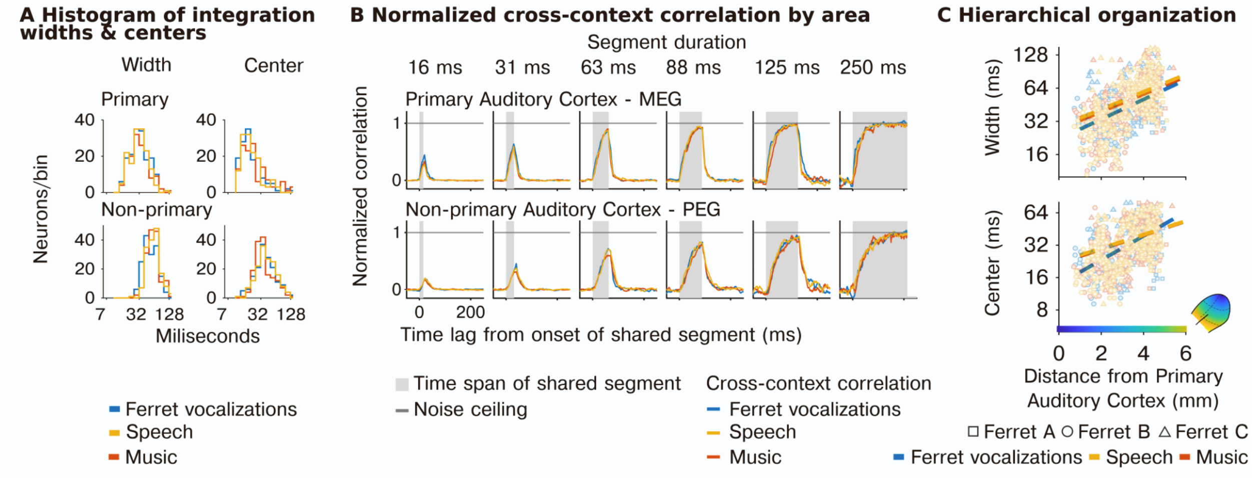

Figure S2. Temporal integration is similar for different stimulus categories. Related to Figure 2.

(A) Histograms of model-fitted integration window parameters (widths and centers) for units in primary and non-primary auditory cortex across the three sound categories tested (ferret vocalizations, speech, music). (B) Normalized median cross-context correlation for primary and non-primary cortex, plotted separately for each sound category. (C) Scatter plots of the model-fitted integration centers and widths as a function of distance to primary auditory cortex computed separately for each category with best-fit lines.

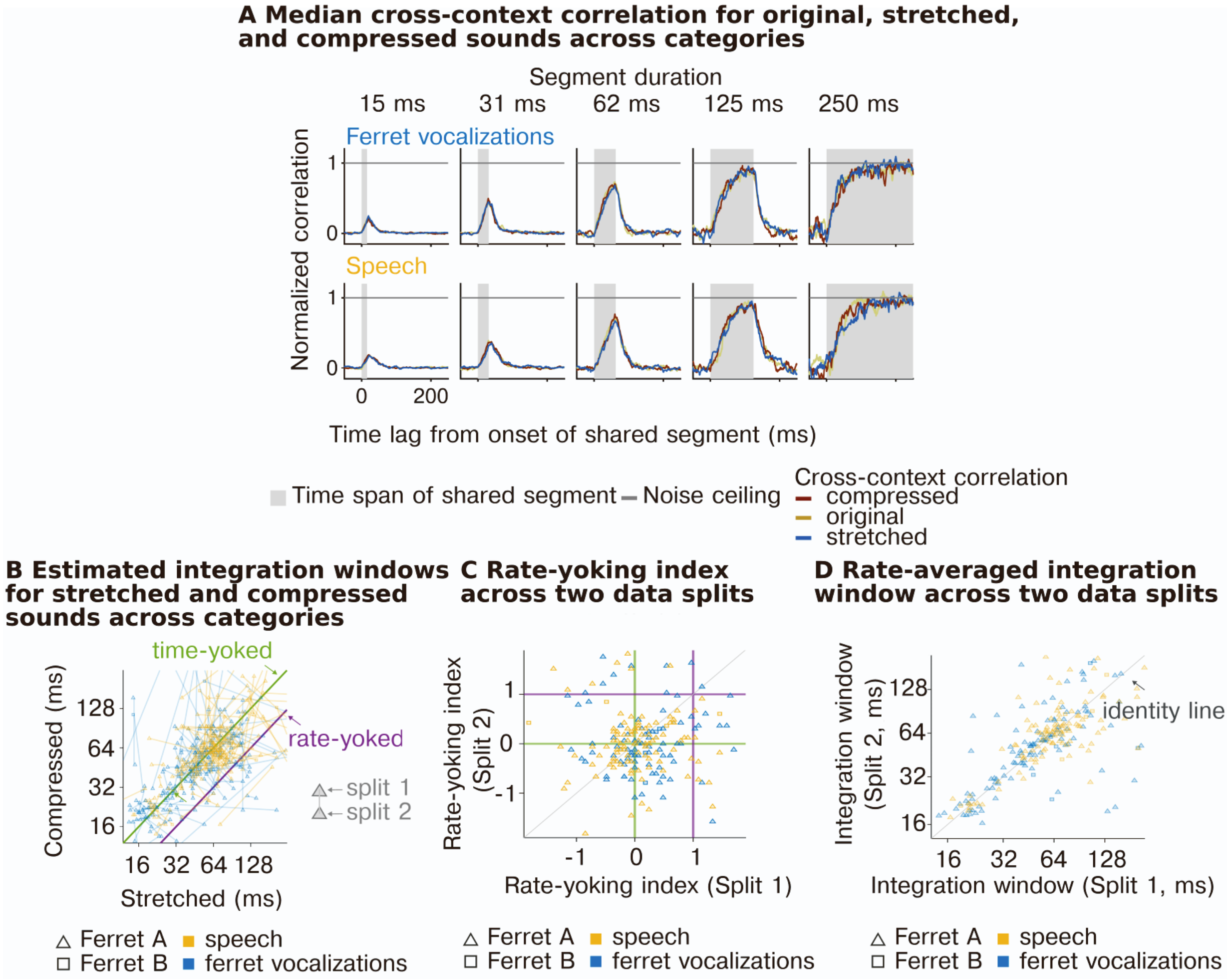

Figure S3. Time-yoked integration is consistent across sound categories. Related to Figure 4.

Format is the same as Figure 4, but plotting results separately for speech and ferret vocalisations. (A) Median normalized CCC across all recorded units for stretched, original, and compressed stimuli computed separately for both sound categories tested (ferret vocalizations and speech). (B) Integration windows (widths) of all units for compressed (x-axis) and stretched (y-axis) stimuli plotted separately for each sound category (speech in yellow and ferret vocalizations in blue). Green and purple lines show the prediction from a time-yoked vs. rate-yoked response. (C) Reliability of rate-yoking index across two independent data splits, separately for each category. (D) Reliability of integration windows averaged across stimuli rates for comparison, again separately for each category.

	US	France
Age	6-9 months	1-3 years
Sex	male	female
Recording type	acute laminar probes	chronic multi-electrode arrays
Sound system	free field central speaker; 30° azimuth controlatéral from the recording site	free field central speaker

Table S1. Summary of the differences between the two experimental sites. Related to STAR Methods.

Columns represent two experimental sites (OSHU in the US and ENS in France). The rows represent differentiating variables.
