# Supplementary material for: Neurons in auditory cortex integrate information within a constrained and context-invariant temporal window
Source: Curr Biol. Author manuscript; Available in PMC 2026 Jul 3. (PMC13331568; doi:10.1016/j.cub.2025.11.011)
Supplement: Supplemental 2 [file NIHMS2179570-supplement-Supplemental_2.pdf]

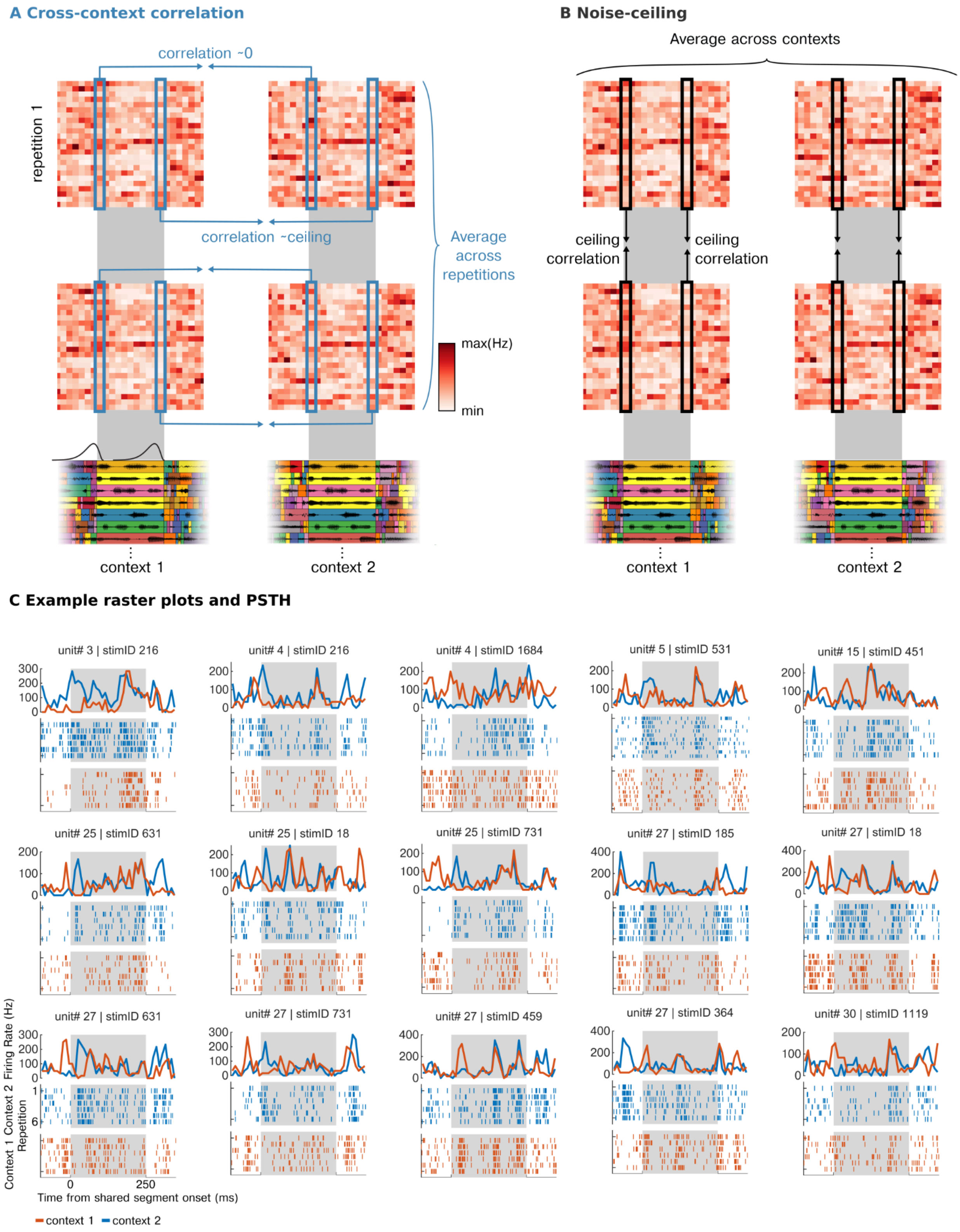
A Cross-context correlation
correlation ~0
repetition 1
correlation ~ceiling
Average across repetitions
max(Hz)
min
context 1
context 2
B Noise-ceiling
Average across contexts
ceiling correlation
ceiling correlation
context 1
context 2
C Example raster plots and PSTH
unit# 3 | stimID 216
unit# 4 | stimID 216
unit# 4 | stimID 1684
unit# 5 | stimID 531
unit# 15 | stimID 451
unit# 25 | stimID 631
unit# 25 | stimID 18
unit# 25 | stimID 731
unit# 27 | stimID 185
unit# 27 | stimID 18
unit# 27 | stimID 631
unit# 27 | stimID 731
unit# 27 | stimID 459
unit# 27 | stimID 364
unit# 30 | stimID 1119
Firing Rate (Hz)
Context 1 Context 2 Repetition
Time from shared segment onset (ms)
context 1
context 2

S1.1. More detailed schematic of the cross-context correlation (A) and noise-ceiling (B) as applied to an example unit and example raster plots showing context invariance in single unit spiking activity. Related to Figure 1.

This legend gives a brief description of the core ideas as they are linked to this figure. See the text for a more in-depth description of the analyses. (A) The neural response timecourse of the unit is reorganised as a matrix. Specifically, the response timecourse surrounding all of the segments of a given duration (500 ms in this schematic) was compiled into a segment-by-time matrix, aligned to segment onset (as in **Figure 1B**). The gray shaded area shows the time period when the shared segment was present. Each row contains the response timecourse to a single segment, and each column contains the response to many segments for a single time lag relative to segment onset. Separate matrices were computed for two different contexts (context 1: left, context 2: right) and two different repetitions of the same context (rep 1: top, rep 2: bottom). Below the matrices, we plot waveforms of corresponding segments: the top waveform corresponds to the segment for the first row of the matrix, the second waveform from the top corresponds to the segment of the second row, and so on. The cross-context correlation is computed by correlating corresponding columns of matrices from different contexts (blue columnar boxes), separately for each repetition, and then averaging the correlation coefficients across repetitions. The correlation is computed separately for each time lag relative to segment onset, and a schematic of the hypothesized integration window at each time lag is shown below, overlaid on the stimulus waveforms. At segment onset, the integration window will fall on the preceding context segments, which are independent across contexts, and the correlation should thus be approximately 0. If the integration window is less than the segment duration, then there will be a moment when the window is fully contained within the shared segment, and at this moment, the cross-context correlation should equal the maximum possible value given by the noise ceiling. One can visually observe that the responses from this unit become more similar as time progresses within the segment. To make it possible to visually observe the key trends in this figure, we used a 50 ms bin. Quantitative analyses were performed using a much smaller bin (5 ms) to ensure that the bin size did not upward-bias the measured integration window. (B) To compute the noise ceiling, we correlated columns across repetitions, separately for each context, and averaged the correlation coefficient across contexts. Because the context is identical, the noise ceiling provides an upper bound of the maximum possible correlation that could be observed when comparing responses across different contexts. (C) Fifteen panels showing examples of context invariance visible in the spiking activity of individual units. Each panel is composed of two subplots showing raster plots for a single shared segment in two different contexts (context 1: blue-top, context 2: bottom-orange) across all repetitions, along with a PSTH (10ms bins), averaged across all repetitions separately for each context. Note how for each cell, the response initially differs between contexts before converging after some delay. Note that some variance is expected across contexts due to noise, which our measures correct for by comparing the cross-context correlation with a ceiling correlation.

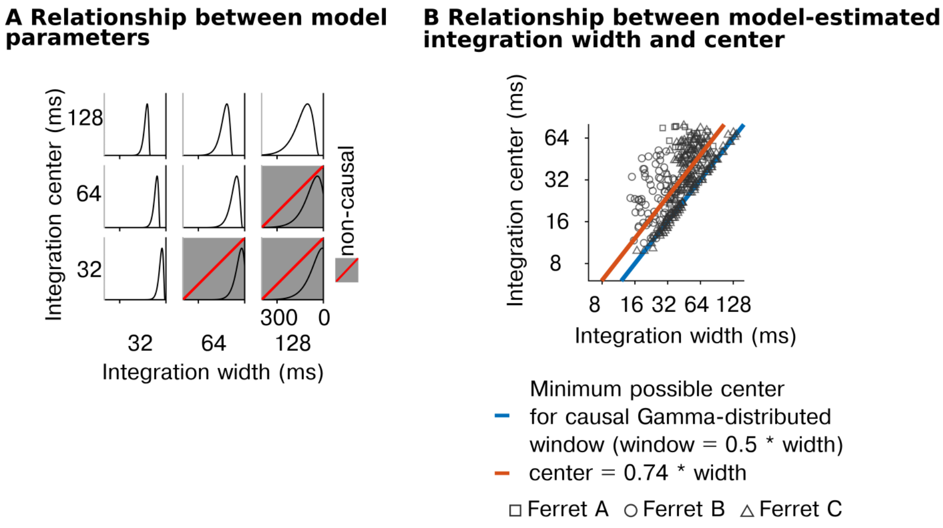

S1.2. Relationship between the center and width of the integration window. Related to Figure 2.

(A) Integration windows were estimated using a parametric window (Gamma distribution) with a varying width and center. We estimated the width and center that best predicted the cross-context correlation, excluding parameters that yielded a non-causal window. This panel plots examples of the parameter window as a function of the window's width (x-axis) and center (y-axis). Combinations of parameters that led to acausal windows were excluded (gray box with red dashed line) because they are not biologically possible. (B) Scatter plot of estimated integration centers vs widths for all units with a best-fit linear function overlaid (orange line). The blue line shows the minimum possible center for a causal, Gamma-distributed window.

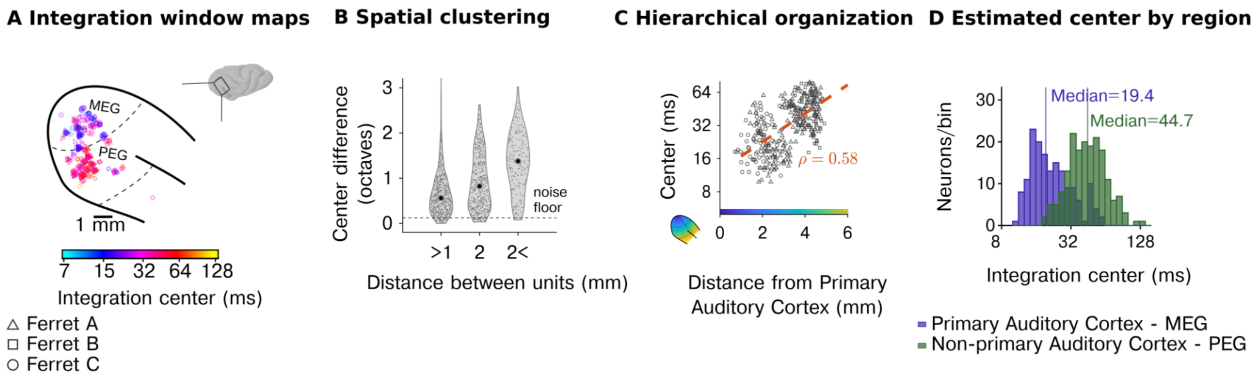

S1.3. Organisation of integration centers across neural populations in the auditory cortex. Related to Figure 2.

(A) Anatomical map of model-estimated integration centers in three animals (window center: median of the interval containing 75% of the window's mass). (B) The difference between the centers of the integration windows between pairs of units as a function of their spatial distance, demonstrating that nearby units have more similar centers. (C) Centers of the integration windows as a function of distance

to the primary auditory cortex (see color map in inset). (D) Histograms of the centers of the integration windows for primary and non-primary auditory cortex showing substantial diversity across units and hierarchical organization.

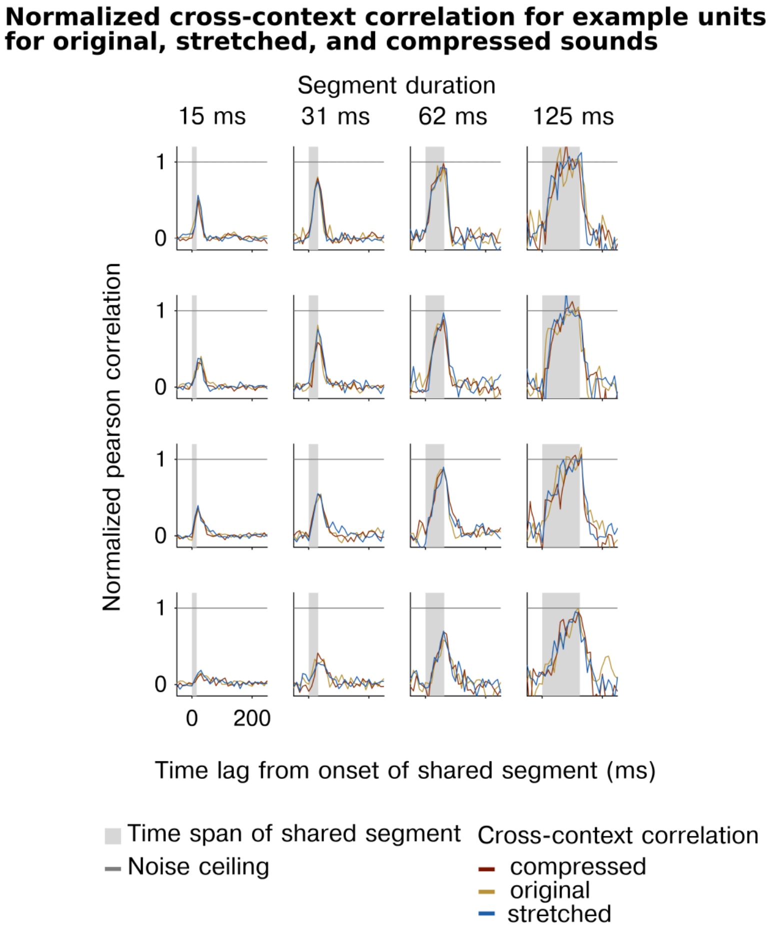

S1.4. Normalized cross-context correlation for example multi-units for different sound rates. Related to Figure 4.

Normalized cross-context correlation for five individual units (rows) plotted separately for each of the three different sound rates (compressed, original, stretched). The normalized CCC was computed by dividing the CCC by the noise ceiling separately for every segment duration and time point.

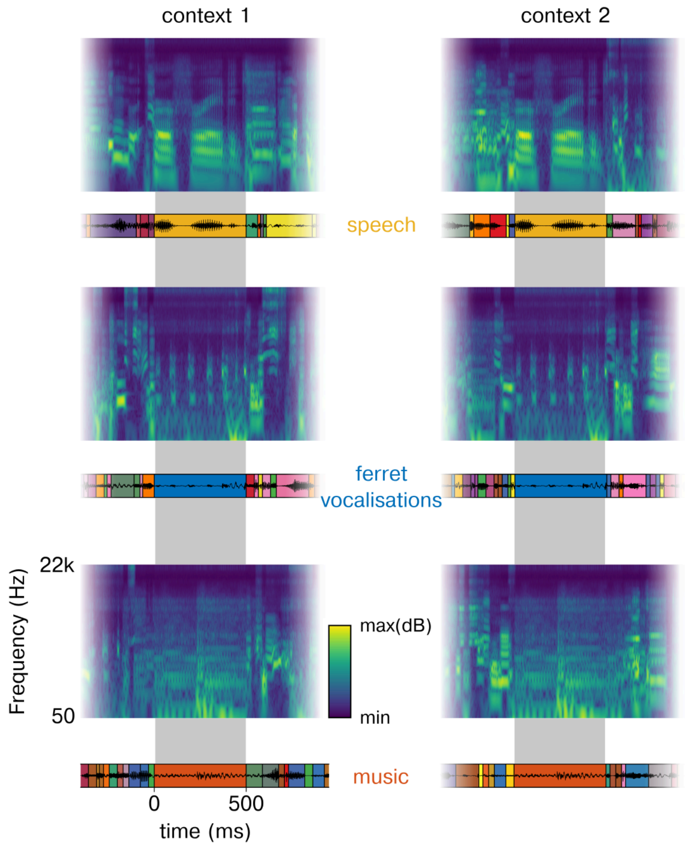

S1.5. Example spectrograms of the segment sequences used in Experiment I. Related to STAR Methods.

Each row shows a different example segment (top: speech segment, middle: ferret vocalisation segment: bottom: music segment). The left and right panels show spectrograms of the same segment in two different contexts.

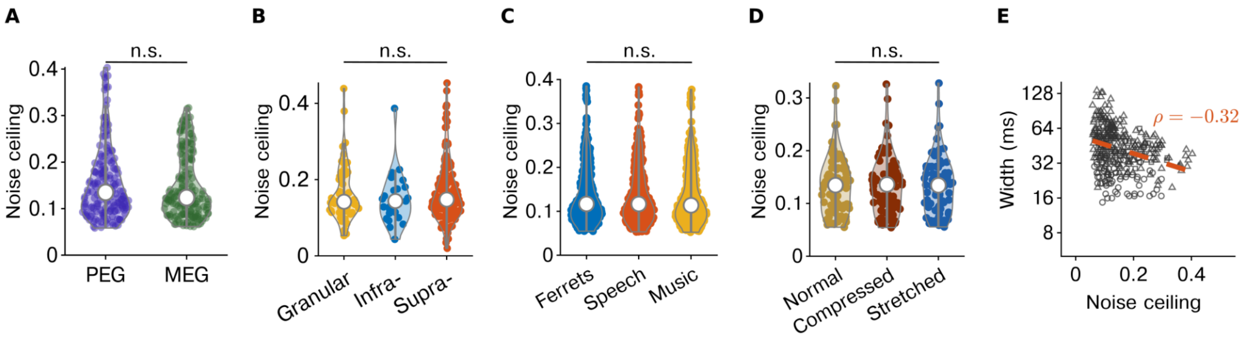

S1.6. Noise ceiling across conditions and regions. Related to STAR Methods.

(A) Histograms of the distribution of the noise-ceiling in the Primary (PEG) and Non-Primary (MEG) auditory cortex. (B) Violin plot of the average noise-ceiling for all units recorded in Experiment I across the sound categories. (C) Violin plot of the average noise-ceiling for all units recorded in Experiment II across the layers. (D) Violin plot of the average noise-ceiling for all units recorded in Experiment III for the 3 experimental conditions. (E) Scatter plot representing the relationship between the noise ceiling and integration width (left) and center (right). Orange lines show affine fits, with Spearman correlation coefficients indicated.

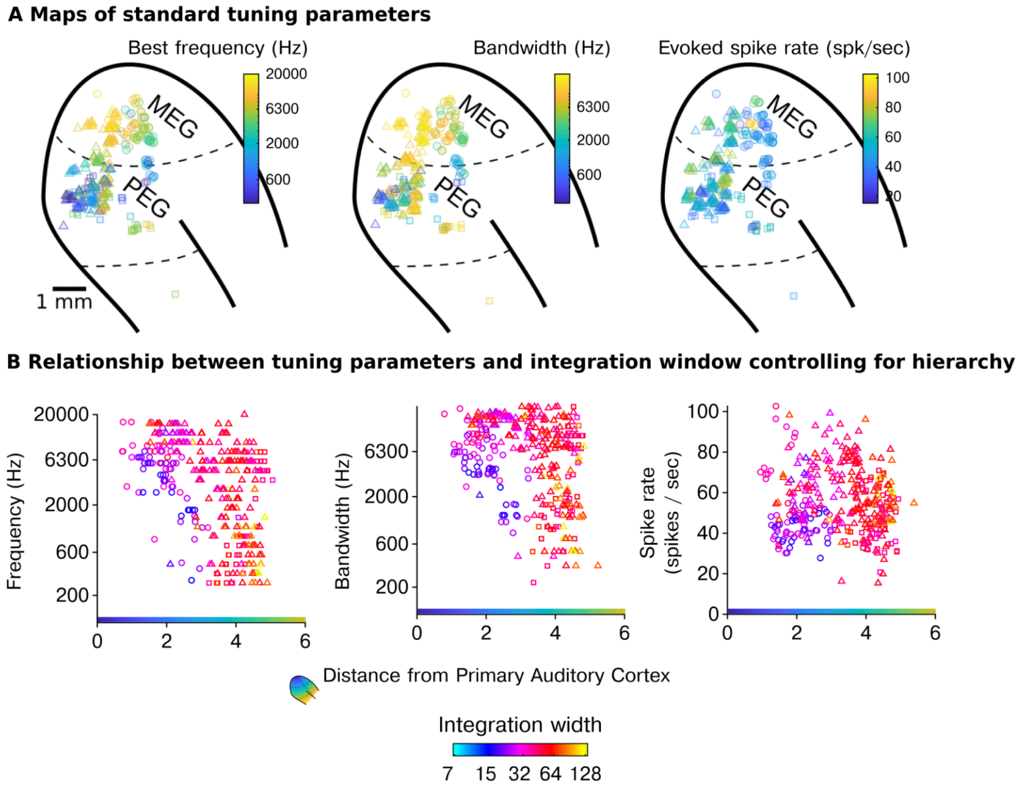

S1.7. Relationship between standard tuning parameters and neural integration window. Related to STAR Methods.

(A) Maps of standard tuning parameters. (B) Scatter plots show the tuning parameters for each cell plotted against a measure of anatomical hierarchy (distance to primary auditory cortex) with the integration window indicated by color.
